# An Innovative Approach to Treat Incisors Hypomineralization (MIH): A Combined Use of Casein Phosphopeptide-Amorphous Calcium Phosphate and Hydrogen Peroxide—A Case Report

**DOI:** 10.1155/2012/379593

**Published:** 2012-11-26

**Authors:** Stefano Mastroberardino, Guglielmo Campus, Laura Strohmenger, Alessandro Villa, Maria Grazia Cagetti

**Affiliations:** ^1^Dental Clinic, San Paolo Hospital, University of Milan, 20142 Milan, Italy; ^2^Dental Institute, University of Sassari, 07100 Sassari, Italy

## Abstract

Molar Incisor Hypomineralization (MIH) is characterized by a developmentally derived deficiency in mineral enamel. Affected teeth present demarcated enamel opacities, ranging from white to brown; also hypoplasia can be associated. Patient frequently claims aesthetic discomfort if anterior teeth are involved. This problem leads patients to request a bleaching treatment to improve aestheticconditions.Nevertheless, hydrogen peroxide can produce serious side-effects, resulting from further mineral loss. Microabrasion and/or a composite restoration are the treatments of choice in teeth with mild/moderate MIH, but they also need enamel loss. Recently, a new remineralizing agent based on Casein Phosphopeptide-Amorphous Calcium Phosphate (CPP-ACP) has been proposed to be effective in hypomineralized enamel, improving also aesthetic conditions. The present paper presents a case report of a young man with white opacities on incisors treated with a combined use of CPP-ACP mousse and hydrogen peroxide gel to correct the aesthetic defect. The patient was instructed to use CPP-ACP for two hours per day for three months in order to obtain enamel remineralization followed by a combined use of CPP-ACP and bleaching agent for further two months. At the end of this five-month treatment, a noticeable aesthetic improvement of the opacities was observed.

## 1. Introduction

“Molar Incisor Hypomineralization” (MIH) is the term used to describe teeth, generally permanent incisors and first molars, characterized by a developmentally derived deficiency in mineral enamel. Affected teeth present demarcated enamel opacities, ranging from white to brown, according to the severity of the disease and the hypoplasia that can be associated.

Hypomineralized enamel is soft and porous and occasionally it undergoes to posteruptive breakdown, resulting in anomalous noncarious cavities [1]. Scanning electron microscope and semiquantitative analysis by Energy Dispersive X-ray Spectrometry showed that affected enamel is not organized in hydroxyapatite crystals and it is low in calcium and phosphate ions [2].

Patients affected by MIH present several clinical problems, including rapid wear, enamel loss, increased susceptibility to caries, and sensitivity [1]. Patients frequently claim aesthetics discomfort when anterior teeth are involved. This problem leads patients to request a bleaching treatment to improve aesthetic conditions. Nevertheless, hydrogen peroxide-based gels applied on enamel produce serious side-effects such as hardness decrease and increased roughness and morphologic alterations resulting from mineral loss [3]. Low concentration bleaching peroxides can change calcium and phosphate mineral content of bleached enamel [4]. These enamel changes may highlight the appearance of opacities. Therefore, enamel bleaching is not safe for hypomineralized enamel low in calcium and phosphate content.

Microabrasion and/or a composite restoration are the treatments of choice to improve aesthetic conditions in teeth with mild/moderate MIH, but this approach needs enamel removal. Furthermore, the adhesion of composite resins to hypomineralized tissue is critical [1]. A conservative approach to improve the conditions of teeth affected by mild/moderate MIH would be useful.

Recently, a new remineralizing agent based on Casein Phosphopeptide-Amorphous Calcium Phosphate (CPP-ACP) (GC Tooth Mousse, GC Corporation 76-1 HASUNUMA-CHO ITABASHI-KU, Tokyo, Japan) has been developed. CPP stabilizes high concentration of calcium and phosphate that bind themselves to pellicle and plaque. This reservoir of ions, under acid challenge, maintains a supersaturated mineral environment, reduces demineralization, and enhances remineralization of enamel [3]. CPP-ACP supplementation has been shown to be effective in remineralization of the enamel affected by MIH, resulting in an aesthetic improvement too [2]. However, this clinical approach requires a long-time treatment that could last months or years, requiring patients' cooperation.

The hypothesis tested in the present paper is that a combined use of CPP-ACP mousse and hydrogen peroxide gel may minimize the aesthetic defect caused by MIH opacities without requiring microabrasion or a composite restoration.

## 2. Case Presentation

**Figure 1:**
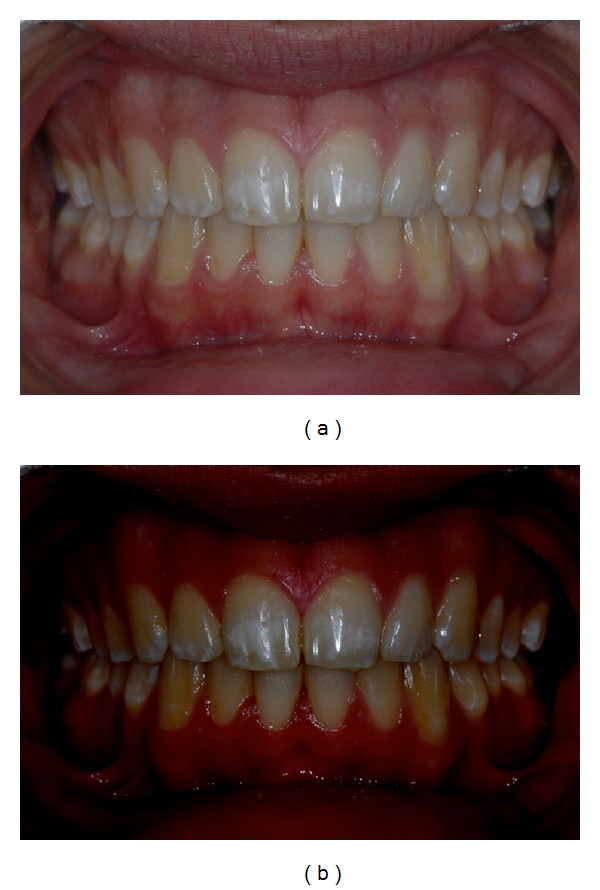
(a) Initial frontal picture. (b) Software-modified initial frontal picture.

**Figure 2:**
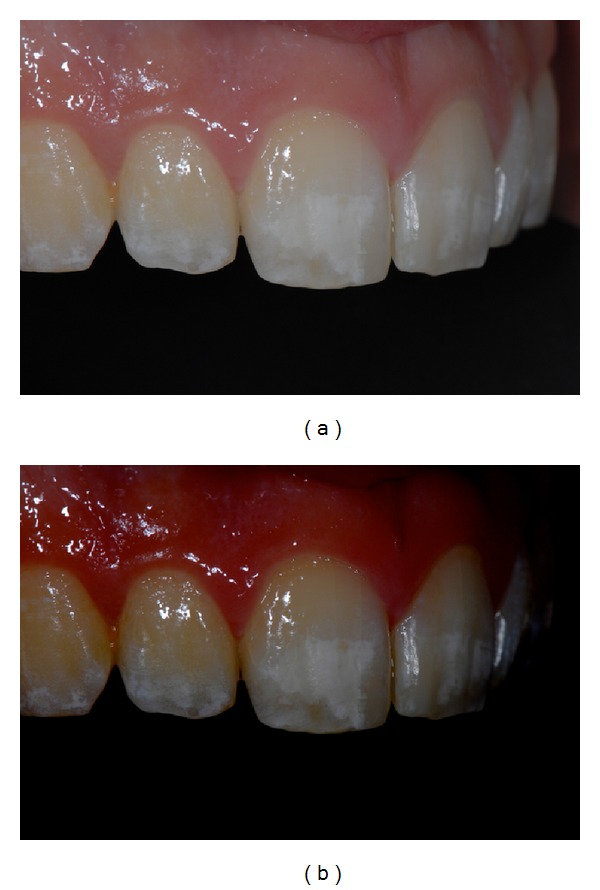
(a) Initial right side picture. (b) Software-modified initial right side picture.

**Figure 3:**
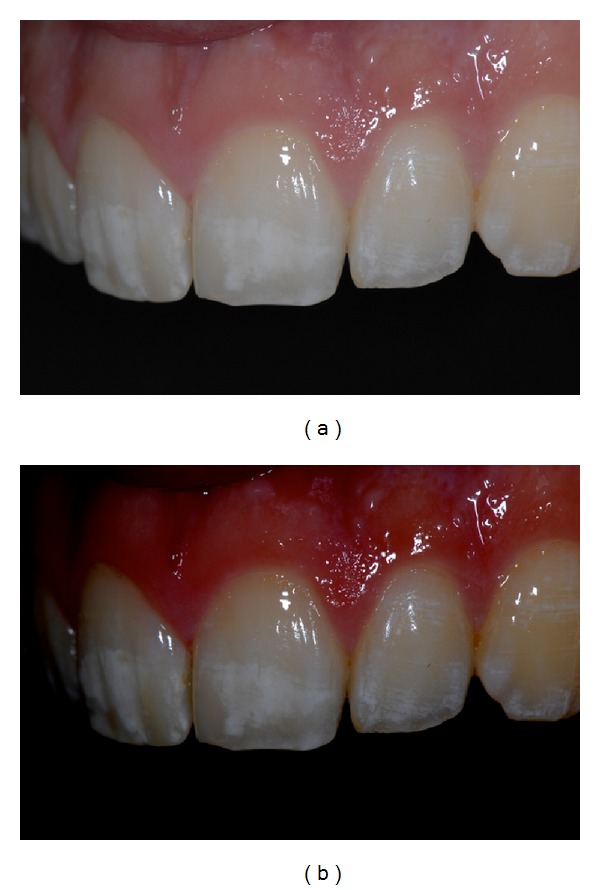
(a) Initial left side picture. (b) Software-modified initial left side picture.

The present paper shows a case report of a seventeen-year-old young man (M. N.) affected by moderate MIH. Superior central incisors show white-demarcated opacities in the incisal half of the labial surfaces. All the other teeth show less extensive incisal and occlusal opacities (Figures 1(a), 2(a), and 3(a)). Dental sensitivity is reported as normal. M. N. states a great aesthetic concern.

Consent was signed by M. N. and his parents: a combined CCP-ACP/hydrogen peroxide individualized protocol was used.

Pictures were captured under standardized conditions and, then, were edited using an imaging software (iPhoto, Apple, CA, USA) only increasing contrast and saturation exclusively in order to enhance the visualization of opacities (Figures 1(b), 2(b), and 3(b)).

Alginate impressions were realized to obtain custom trays using low-density polyethylene plates in a vacuum plasticizer.

The patient together with custom trays received a GC Tooth Mousse tube and he was instructed to use the trays two hours/day with CPP-ACP inside, after an accurate toothbrushing. This first step lasted for three months, in order to obtain a noticeable remineralization of the enamel opacities. Dental examination was performed every month, in order to enhance M. N.'s motivation and take pictures.

After a three-month period, a combined use of CPP-ACP and bleaching agent was applied. A low-concentration hydrogen peroxide product was used. A 20% carbamide peroxide gel (Opalescence 20% Whitening gel, Ultradent Products, Inc. 505 West 10200 South, South Jordan, UT 84095, USA), approximately an equivalent to 6% of hydrogen peroxide, was provided.

The patient was instructed to wear trays with the bleaching agent for two hours on two consecutive days and for the remaining five days of the week the remineralizing agent. This protocol was repeated for two months in order to obtain 14 days of bleaching treatment (according to the manufactures instructions) (Table 1). During these two months, dental examinations were weekly performed to ensure safety and efficacy of the treatment and to early intercept any side-effects of hydrogen peroxide on MIH.

**Figure 4:**
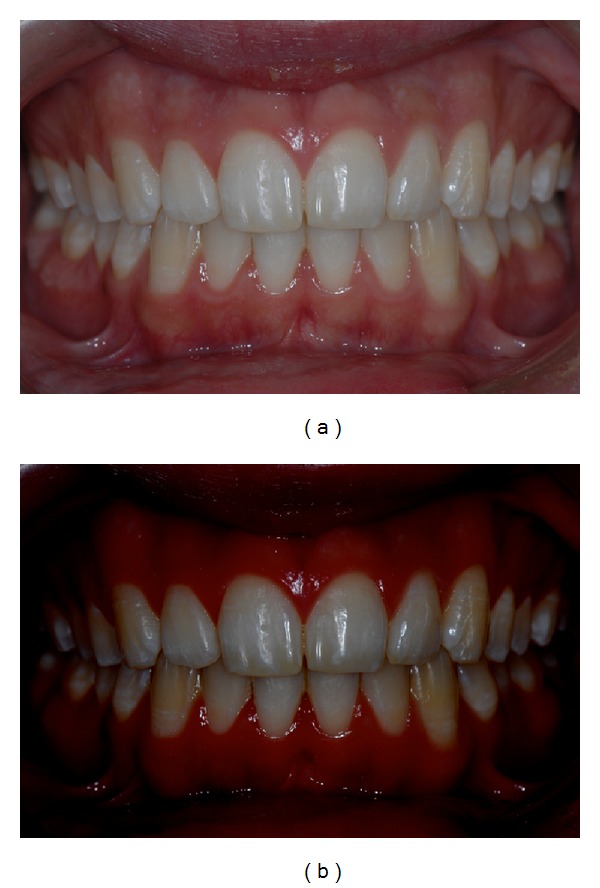
(a) Final frontal picture. (b) Software-modified final frontal picture.

**Figure 5:**
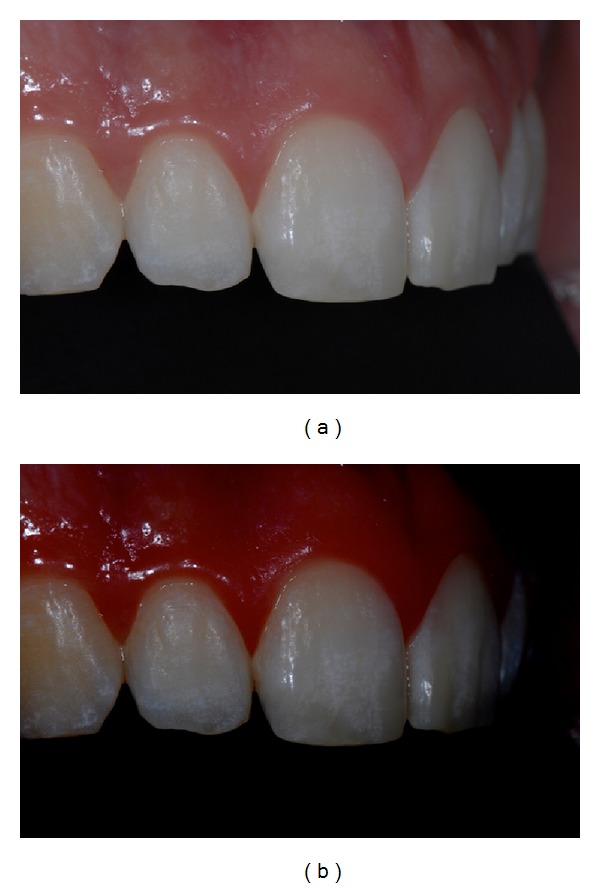
(a) Final right side picture. (b) Software-modified final right side picture.

**Figure 6:**
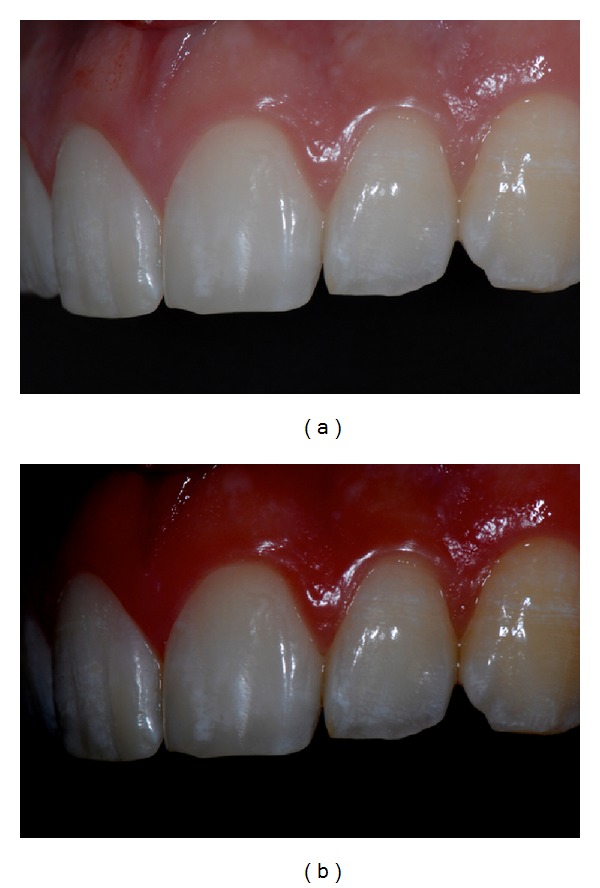
(a) Final left side picture. (b) Software-modified final left side picture.

At the end of this five-month treatment, a noticeable aesthetic improvement of opacities was observed (Figures 4(a), 5(a), 6(a), 4(b), 5(b), and 6(b)).

## 3. Discussion

MIH is a widespread dental condition; its prevalence ranges from 2.4% to 40.2% in different studies [5]. It is likely that MIH is not caused by one specific aetiological factor. An increased risk of MIH occurs if several agents or conditions act synergistically [5]. These factors act systemically and affect the developmental enamel during prenatal, perinatal, or the childhood period [1]. Particularly, pathogenic “noxae” during the first year of life are more frequent in children with MIH. Asthma, pneumonia, upper respiratory tract infections, otitis media, antibiotics, dioxins in mother's milk, tonsillitis and tonsillectomy, and exanthematous fevers during childhood were involved in the process [6]. Amoxicillin has been shown to affect enamel formation in animal model and a twofold increase in risk of MIH has been recorded if amoxicillin was used during the first year [5]. So, at the present time, the aetiology of MIH still remains unclear.

Clinical problems associated with MIH regard function and aesthetics. Treatment approach for teeth affected by MIH is wide, ranging from prevention, restoration, to extraction [1]. MIH forms need a noninvasive treatment in order to preserve enamel integrity; nevertheless, simple and unambiguous clinical guidance is missing nowadays.

MIH opacities have a total reduced mineral content (about 20% of the weight) and a relative increase in organic substance [2, 7, 8]; its remineralization with conventional or new aids, like fluoride or CCP-ACP, is an arduous and slow proceeding. Microabrasion and/or composite resin restorations are the most used solutions to improve aesthetics of anterior teeth affected by opacities, but these approaches are quite invasive.

The use of bleaching systems on hypomineralized teeth is not recommended because of mineral changes caused by peroxides especially on enamel presenting an increase in carbon content and a decrease in calcium and phosphate content [2]. During a bleaching treatment, peroxides initiate oxide-reduction reaction that may lead to dissolution of both organic and inorganic matrices [9]. Enamel remineralization from saliva action is expected; nevertheless, an increase of enamel porosity, depression, superficial irregularities [10], roughness [11], and a decrease of hardness [12] are reported. This hardness reduction could be related to mineral loss resulting from demineralization [13].

CCP-ACP containing paste applied on teeth before and after the bleaching treatment [9], or mixed in similar proportion with the bleaching gel [3], has shown to be able to prevent roughness and hardness decrease of bleached teeth, without interfering with bleaching effect. CCP-ACP has, also, been shown to be effective in remineralization of MIH opacities *in vivo*, helping functional restorative techniques in MIH molars and aesthetics in untreated incisors [2]. Up to date it has never been investigated the possibility to treat MIH opacities using bleaching system combined with CCP-ACP mousse.

The protocol used in this paper has shown to be effective to improve aesthetics, although some weaknesses are to be considered. A long-term patient collaboration is required: two hours per day for five consecutive months. Furthermore, this protocol was applied to a patient affected by a moderate MIH; upper frontal incisors were the teeth mostly involved. White-demarcated opacities were wide, involving the incisal half of the labial surfaces and hypoplasia was not associated.

The short follow-up period (two months) could represent a limit. Results seem to be stable, but they have to be monitored for a longer period of time. In order to decrease the risk of recurrence of the defects, a maintenance therapy with CCP-ACP, (i.e., one time/week) should be considered.

This protocol has been described in the present paper for the first time and further corrections or revisions could be necessary. As recently described by de Vasconcelos et al. [3], in an *in vitro* study, the possibility to mix in similar proportion CCP-ACP mousse and the bleaching gel could be taken into account in enamel opacities treatment, decreasing the bleaching period to the usual two/three weeks.

An important aspect that must be considered is the need to personalize the protocol on the features of the opacities and the response of the defect to the treatment. The combined use of hydrogen peroxide and CCP-ACP, for example, could be performed with a ratio range from 1 : 6 to 3 : 4, depending on the opacity response to the bleaching agent.

The present protocol based on the combined use of CPP-ACP and hydrogen peroxide has shown to be effective and safe in bleaching teeth with enamel white opacities, allowing to preserve enamel integrity without recurring to restorative treatment or enamel abrasion.

## Figures and Tables

**Table 1:** Scheme of the combined use of Casein Phosphopeptide-Amorphous Calcium Phosphate (CCP-ACP) and hydrogen peroxide.

	Month				
	I	II	III	IV	V
	Product utilization days/week				
CCP-ACP 2 hrs/day	7	7	7	5	5
Hydrogen peroxide 2 hrs/day	/	/	/	2	2

## References

[1] Lygidakis NA (2010). Treatment modalities in children with teeth affected by molar-incisor enamel hypomineralisation (MIH): a systematic review. *European Archives of Paediatric Dentistry*.

[2] Baroni C, Marchionni S (2011). MIH supplementation strategies: prospective clinical and laboratory trial. *Journal of Dental Research*.

[3] de Vasconcelos AA, Cunha AG, Borges BC (2012). Enamel properties after tooth bleaching with hydrogen/carbamide peroxides in association with a CPP-ACP paste. *Acta Odontologica Scandinavica*.

[4] Efeoglu N, Wood D, Efeoglu C (2005). Microcomputerised tomography evaluation of 10% carbamide peroxide applied to enamel. *Journal of Dentistry*.

[5] Lygidakis NA, Wong F, Jälevik B, Vierrou AM, Alaluusua S, Espelid I (2010). Best clinical practice guidance for clinicians dealing with children presenting with molar-incisor-hypomineralisation (MIH): an EAPD policy document. *European Archives of Paediatric Dentistry*.

[6] Willmott NS, Bryan RA, Duggal MS (2008). Molar-incisor-hypomineralisation: a literature review. *European Archives of Paediatric Dentistry*.

[7] Jälevik B, Norén JG (2000). Enamel hypomineralization of permanent first molars: a morphological study and survey of possible aetiological factors. *International Journal of Paediatric Dentistry*.

[8] Farah RA, Swain MV, Drummond BK, Cook R, Atieh M (2010). Mineral density of hypomineralised enamel. *Journal of Dentistry*.

[9] Gama Cunha AG, Meira de Vasconcelos AA, Dutra Borges BC (2012). Efficacy of in-office bleaching techniques combined with the application of a Casein Phosphopeptide-Amorphous Calcium Phosphate paste at different moments and its influence on enamel surface properties. *Microscopy Research and Technique*.

[10] Ferreira SS, Araújo JLN, Morhy ON, Tapety CMC, Youssef MN, Sobral MAP (2011). The effect of fluoride therapies on the morphology of bleached human dental enamel. *Microscopy Research and Technique*.

[11] Martin JM, de Almeida JB, Rosa EA (2010). Effect of fluoride therapies on the surface roughness of human enamel exposed to bleaching agents. *Quintessence International*.

[12] Borges AB, Samezima LY, Fonseca LP, Yui KCK, Borges ALS, Torres CRG (2009). Influence of potentially remineralizing agents on bleached enamel microhardness. *Operative Dentistry*.

[13] Featherstone JD, ten Cate JM, Shariati M, Arends J (1983). Comparison of artificial caries-like lesions by quantitative microradiography and microhardness profiles. *Caries Research*.

